# Visual perspective and body ownership modulate vicarious pain and touch: A systematic review

**DOI:** 10.3758/s13423-024-02477-5

**Published:** 2024-03-01

**Authors:** Matteo P. Lisi, Martina Fusaro, Salvatore Maria Aglioti

**Affiliations:** 1 CLN2S@Sapienza, Fondazione Istituto Italiano di Tecnologia (IIT) and Department of Psychology, Sapienza University of Rome, Viale Regina Elena 291, 00161 Rome, Italy; 2 IRCCS, Santa Lucia Foundation, Via Ardeatina 306, 00179 Rome, Italy

**Keywords:** Vicarious pain and touch, Systematic review, Visual perspective, Body ownership, Immersive virtual reality

## Abstract

We conducted a systematic review investigating the influence of visual perspective and body ownership (BO) on vicarious brain resonance and vicarious sensations during the observation of pain and touch. Indeed, the way in which brain reactivity and the phenomenological experience can be modulated by blurring the bodily boundaries of self-other distinction is still unclear. We screened Scopus and WebOfScience, and identified 31 articles, published from 2000 to 2022. Results show that assuming an egocentric perspective enhances vicarious resonance and vicarious sensations. Studies on synaesthetes suggest that vicarious conscious experiences are associated with an increased tendency to embody fake body parts, even in the absence of congruent multisensory stimulation. Moreover, immersive virtual reality studies show that the type of embodied virtual body can affect high-order sensations such as appropriateness, unpleasantness, and erogeneity, associated with the touched body part and the toucher’s social identity. We conclude that perspective plays a key role in the resonance with others' pain and touch, and full-BO over virtual avatars allows investigation of complex aspects of pain and touch perception which would not be possible in reality.

## Introduction

### Vicarious pain and touch

Empathy is commonly defined as the ability to consciously share and understand the affective state of another individual, who is recognized as source of that state, and to generate a state similar or identical to the one perceived or imagined in others (De Vignemont & Singer, 2006). Neuroscientific accounts of empathy propose that observing another person’s state activates overlapping brain regions in the observer, providing us with an embodied simulation of their feelings (De Waal & Preston, 2017; Gallese, 2003; Goldman, 2006). From an evolutionary perspective, vicarious brain resonance (i.e., overlapping brain activity in the sensory regions during both perception and observation of a given stimulus) may represent a foundational layer of empathy, which is shared at the phylogenetic level between humans and other animals (De Waal, 2008; Langford et al., 2006), and at the ontogenetic level between adults and infants (Bandstra et al., 2011; Decety et al., 2008). Interestingly, overlapping brain activations between personal experience and observation have been shown not only for emotional expressions (Pfeifer et al., 2008) but also for sensory experiences, such as “flesh and bone” pain (Avenanti et al., 2005, 2006) and touch (Blakemore et al., 2005; Keysers et al., 2004). This vicarious brain activity is thought to fundamentally contribute to the somatic experience of others’ sensations, which may range from an automatic and unconscious process through an overt experience of the sensation observed in the other person (Fitzgibbon et al., 2014). Crucially, vicarious resonance is not a static property, but it is affected by individual dispositions and contextual variables, such as the social categorization of the observed other (Avenanti et al., 2010; Ionta et al., 2020) playing an important role in the unfolding of social interactions. The degree to which we resonate with others is indeed associated with our prosocial decision-making (Gallo et al., 2018), while a deficit in vicarious resonance can be associated with social cognition disorders, such as psychopathy (Meffert et al., 2013).

Pain is defined as “an unpleasant sensory and emotional experience associated with, or resembling that associated with, actual or potential tissue damage” (Raja et al., 2020). Evidence suggests that sensory-discriminative components (such as location, duration, and intensity) are mapped onto the sensorimotor cortices (Bingel et al., 2004; Craig, 2003; Porro et al., 1998), while the affective-motivational components (unpleasantness) are coded in several limbic areas including anterior cingulate cortex and anterior insula (Ploner et al., 1999), forming a complex neural network, commonly referred as the “pain matrix” (Ingvar, 1999; Legrain et al., 2011; Peyron et al., 2000; Rainville, 2002). In a well-known functional magnetic resonance imaging (fMRI) study, Singer et al. (2004) demonstrated that the anterior insula and the anterior cingulate cortex were activated both during actual pain and during the imagination of the pain of loved others (Singer et al., 2004), as though only the affective node of the pain matrix was involved in vicarious pain. Further studies confirmed the affective network activity during the observation of painful stimulation on others (Lamm et al., 2011; Morrison et al., 2004). Tellingly, however, the somatosensory components of processing another’s pain were highlighted in a transcranial magnetic stimulation (TMS) study showing that the corticospinal motor representation of hand muscles of individuals observing painful events was specifically inhibited during the direct observation of pain on others (Avenanti et al., 2005). This evidence was further investigated and confirmed in many studies where participants had to observe videos or pictures of others receiving painful stimulation (Avenanti et al., 2009; Betti et al., 2009; Bufalari et al., 2007; Fan & Han, 2008; Minio-Paluello et al., 2009). In a similar vein, Keysers and colleagues (2004) evidenced the existence of a network that is active when observing touch on others. In particular, using fMRI, they showed that observing someone else’s legs being touched with a stick resulted in neural activity in the secondary somatosensory cortex, which is also active during actual touch (Keysers et al., 2004). One year later, Blakemore revealed that the observation of touches was associated with activity both in the primary and secondary somatosensory cortex (Blakemore et al., 2005). Additionally, the authors described a participant reporting conscious feelings of being touched during touch observation and who exhibited vicarious somatosensory activations that were abnormally higher compared to other participants. The investigation of this visuo-tactile type of synaesthesia was later investigated by several studies (see section *Mirror-touch and mirror-pain synaesthesia*). Interestingly, stroking of a hand or a brush on the skin at a velocity range between 3 to 10 cm/s is considered pleasant and can activate a group of fibers, named C tactile (CT, Löken et al., 2009). fMRI studies show that CT-targeted stroking is associated with activations of posterior insular cortex (Olausson et al., 2002, 2010; Olausson et al., 2008) and a network of areas involved in social perception and social cognition, including right posterior superior temporal sulcus, the medial prefrontal cortex, and amygdala (Gordon et al., 2013). According to the “skin as a social organ” hypothesis (Morrison et al., 2010), the feelings of pleasantness coded by the specialized CT-touch pathway may promote homeostatic and affective regulation since the caregiver-infant relationship (Fairhurst et al., 2014), ultimately playing a fundamental role in the building of self and social bonds between individuals (Fini et al., 2023). Different studies have also been conducted investigating whether the observation of pleasant touch on others might elicit in the observer distinct sensations and brain reactivity. What has been observed is that the maximum pleasantness of a pleasant touch matches the velocity needed to activate the CT receptors (Morrison et al., 2011). Moreover, the reported pleasantness is coupled with an activation in the posterior insula, as for the perception of an actual pleasant touch. This selective reactivity to pleasant touch in the insula might be important for individuals to decode for affective values even when just observing stimuli on others.

### Mirror-touch and mirror-pain synaesthesia

A peculiar condition in which the observation of touch elicits first-hand sensations in the observer is *mirror-touch synaesthesia*. Mirror-touch synaesthesia occurs when the observation of tactile stimulation to another leads the synaesthete to report an experience equivalent to being touched (e.g., Blakemore et al., 2005). Similarly, mirror-pain synaesthesia occurs when the observation of noxious stimulation to another induces one to experience pain (e.g., see Giummarra & Bradshaw, 2008). These conditions might occur developmentally or be acquired after a trauma. Two alternative, but not mutually exclusive, accounts have been proposed to explain this condition: Firstly, the *Threshold-theory* posits that mirror-touch and mirror-pain synaesthesia are a result of the overactivation in the somatosensory regions (Blakemore et al., 2005) and they can be induced in neurotypical participants via noninvasive brain stimulation of the primary somatosensory cortex (Bolognini et al., 2013). Secondly, the *Self-Other Theory* highlights the role of atypical self-other distinction, which may result in the self-attribution of others’ experiences (Banissy et al., 2009). Both claims are supported by the evidence that synaesthetes (also defined in the literature as “vicarious responders”; Bowling et al., 2019) display structural differences compared to neurotypical participants, including increased grey matter of somatosensory cortices (Holle et al., 2013) and reduced volume of the right temporoparietal junction, a key node of self-other distinction (Grice-Jackson et al., 2017a, 2017b). In a unifying view, the atypical self-other processing (i.e., less defined borders between self and other) may act as a gating mechanism for enhanced somatosensory processing. This in turn could lead to conscious vicarious experiences of tactile or painful events (Bufalari et al., 2015; Santiesteban et al., 2015; Ward & Banissy, 2015).

### Physical self-other distinction: From visual perspective taking to illusory body ownership

The role of self-other distinction in vicarious pain and touch has also been extensively investigated in neurotypical populations, based on the assumption that attributing the source of the vicarious feelings is a fundamental component of empathy, allowing one to understand the other without the risk of self-other misattribution (De Vignemont & Singer, 2006). Taking the perspective of someone else can occur not only at the cognitive level (i.e., imagining feeling the same state observed or imagined in others or imagining the feelings of others in a given state), but also at the perceptual level (i.e., seeing the state of a model in first vs. third person perspective; Chiu & Yeh, 2018). In desktop-based studies, visual perspective is generally manipulated by rotating the model observed on the screen (Bucchioni et al., 2016). Assuming an egocentric perspective fundamentally contributes to the sense of body ownership (BO), the immediate and pre-reflexive sense that the body belongs to us. BO is considered the cornerstone of the sense of self (Gallagher, 2000), and it is assumed to be a result of the integration between different sensory modalities (Ehrsson, 2020) operating in an egocentric reference frame (Petkova et al., 2011). An illusory sense of BO (BOI) over dummy limbs can be induced through experimental manipulations, such as the Rubber Hand Illusion (RHI). In the RHI, participants observe a dummy hand in front of them while the real hand, hidden from view, is stroked. The BOI is stronger when both hands are stroked synchronously compared to the asynchronous stroking condition (Botvinick & Cohen, 1998). Similarly, congruent visuotactile stimulation can also lead to the embodiment of other body parts, as in the case of the enfacement paradigm (Bufalari et al., 2014; Minio-Paluello et al., 2020; Porciello et al., 2018; Sforza et al., 2010). These paradigms allow one to modulate the degree of BOI toward the fake body part and measure subsequent vicarious perception of pain and touch on it.

Research has also focused on developing experimental paradigms to trigger BOI not only over single body parts but also fake humanoid bodies. A fake humanoid body can be presented by an Immersive Virtual Reality (IVR) head-mounted display that occludes any visual information of the real body and displays a fake body in the same posture and location as the real one, inducing what is called a Full Body Ownership Illusion (FBOI) over a mannequin (Petkova & Ehrsson, 2008) or a virtual humanoid avatar (Slater et al., 2010). The congruent visuo-proprioceptive information about the real and the fake body is a necessary and sufficient condition to induce a strong feeling of BOI (Maselli & Slater, 2013), which can be further enhanced by applying a congruent visuotactile stimulation or providing a visuomotor feedback of the participant’s movements. Evidence shows that BOI can be induced on bodies of different age, gender, and ethnicity, and that change implicit biases (Farmer et al., 2014; Peck et al., 2013). The degree to which BOI inductions can affect vicarious resonance and vicarious perceptions remains an open question.

### Objectives

The main aim of this work was to systematically review the evidence on the role of viewing perspective and BOI on vicarious resonance and vicarious perception of pain and touch. Considering the comparatively limited number of studies on the topic, the heterogeneity of the experimental paradigms, and the measured outcomes, we opted for a qualitative synthesis of the results. The variety of outcomes extracted in this review include autonomic and neuroimaging techniques, psychophysical procedures (such as tactile detection paradigms), explicit ratings regarding the intensity and the quality of the observed stimuli, and explicit reports of vicarious (MTS-like) experiences. In this review we test whether (i) the alteration of bodily self-other distinction via the manipulation of visual perspective and body ownership can affect vicarious somatosensory resonance and vicarious perception in neurotypical participants, and (ii) whether mirror-touch and mirror-pain synesthesia are associated with altered body ownership. We used a systematic approach to identify the articles that focused on this relationship. In the first section, we describe the desktop-based paradigms manipulating the visual perspective from which the pain and/or touch stimuli are observed. We then proceed to review the evidence coming from the RHI paradigm, and how the manipulation of different multisensory signals during the induction of the BOI modulates subsequent vicarious perception in neurotypical people and synaesthetes. Finally, we describe the recent literature investigating vicarious pain and touch using the FBOI in IVR.

## Methods

### Transparency and openness

This review followed the PRISMA 2020 guidelines for systematic reviews (Page et al., 2021). The review was not preregistered and a review protocol was not written before starting the review process.

### Eligibility criteria

Searches were limited to human samples and articles published in English. We included studies published in peer-reviewed journals since 1 January 2000.

We were interested in reviewing studies investigating the effect of visual perspective and body ownership on vicarious pain and touch. The studies had to manipulate at least one between-visual perspective or body ownership and investigate vicarious pain and/or touch perception. Studies employing a real somatosensory stimulation were included only when isolating the activity evoked by the visual information. We excluded studies that focused only on cognitive perspective-taking, in which self-other distinction was manipulated by asking participants to imagine being the target of the stimulation. We excluded studies investigating first-hand perception of pain and touch. We excluded qualitative studies and opinion pieces. Moreover, studies employing the display of emotional facial expressions but not somatosensory stimuli were excluded.

Studies were grouped according to the type of experimental setup: desktop-based studies investigating the role of visual perspective by rotating the orientation of the model, paradigms inducing BOI over body parts (including RHI and enfacement), and paradigms inducing full-BOI through IVR.

### Information sources

We performed separate and identical literature searches on Scopus and Web of Science on 22 September 2022 to retrieve studies concerning the effect of visual perspective and body ownership on vicarious pain and/or touch. The same search strategy and terms were employed on Scopus and Web of Science. We performed additional searches by checking the reference list of eligible studies and using Google Scholar.

### Search strategy

We developed the following search terms related to vicarious somatosensory processing and body ownership: (("Vicarious Pain" OR "Vicarious Touch" OR "observ* Pain" OR "observ* touch" OR "Empathy for Pain" OR "Empathy for Touch" OR "Illusory pain" OR "illusory touch") AND (perspective OR ownership OR embod* OR self)).

## Results

### Study selection

**Fig. 1:**
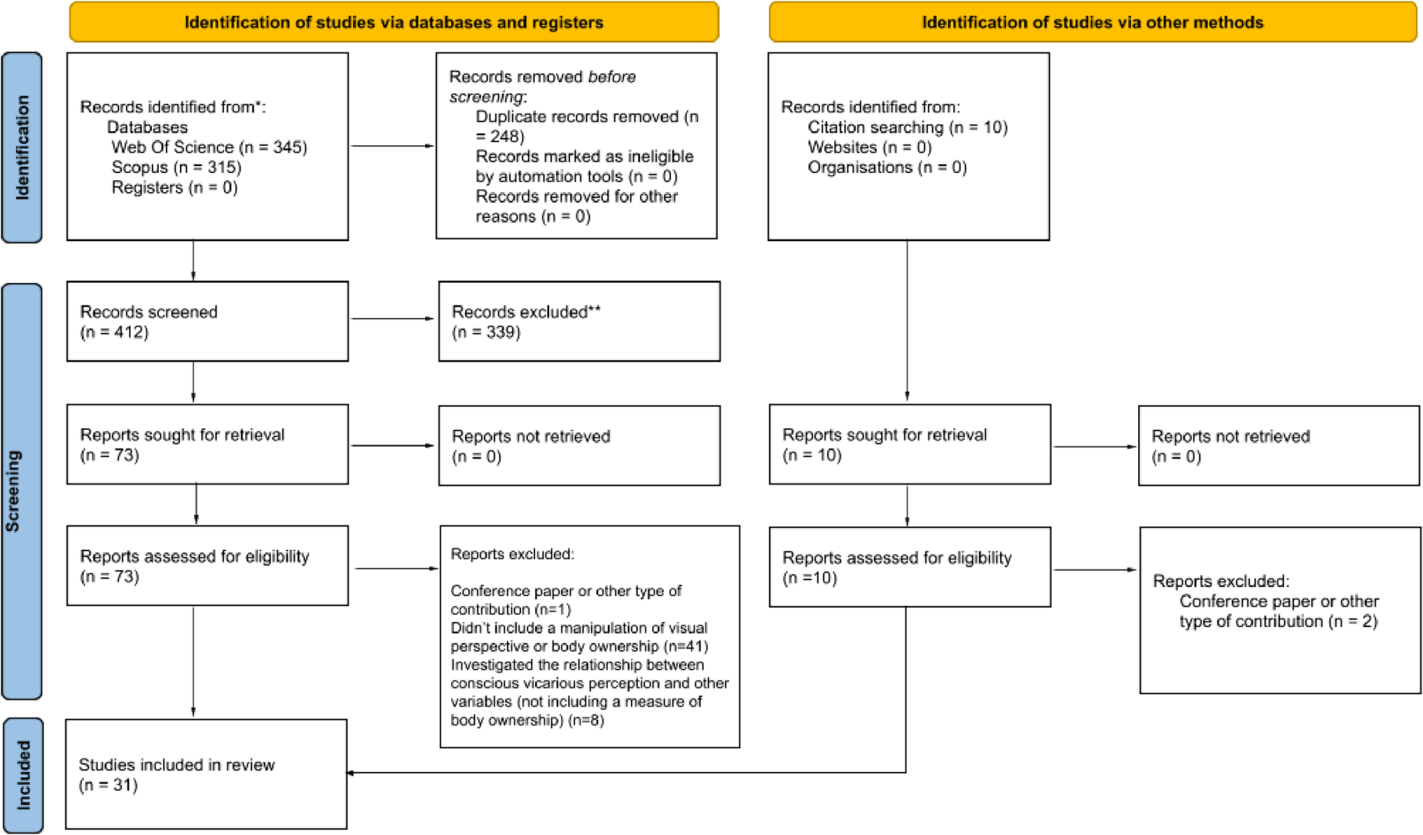
Prisma flow diagram summarizing the selection procedure of the studies included in the review

For more detailed information about the search and screening process, see the PRISMA flow diagram reported in Fig. 1. A total of 345 citations were identified from Scopus and 315 citations from Web of Science. Before the screening, a total of 248 duplicates were removed. Based on the title and the abstract, 339 were excluded, with 73 full-text articles to be retrieved and assessed for eligibility. The main text of these studies was examined to further determine their eligibility according to the predefined criteria. Moreover, ten additional studies were retrieved from other sources (Google Scholar and citation searching), and two of them were excluded for not being peer-reviewed yet. Ultimately, we selected 31 studies, 13 of which were informative concerning the role of visual perspective, 11 about the role of BOI over fake limbs, and seven related to the effect of FBOI in IVR.

### Study characteristics

In Table 1 we reported the following characteristics for each study: sample size details (size, number of females, mean age ± standard deviation, and age range when available); the type of visual perspective (including the rotation degrees employed for 3PP stimuli) or BOI manipulation; whether vicarious pain and/or touch was investigated; the type of behavioral measures and whether the study employed physiological and/or neural measures and techniques; a description of the effects, specifying the contrast of interest and the effect size (eta-squared (*η*^*2*^), partial eta squared (*η*^*2*^*p*), Cohen’s d (*d*), standardized Cohen’s d (*d*_*z*_), Pearson’s R (*r*), Pearson’s R-squared (*R*^*2*^), marginal R-squared (*R*^*2*^*m*), conditional R-squared (*R*^*2*^*c*) found in the study. The characteristics and the findings of the studies are discussed in the *Literature review* section below. Thirteen studies used a manipulation of physical perspective (section A). Eleven studies induced an illusion of embodiment over a fake limb. Specifically, five studies investigated how multisensory stimulation during the body ownership induction affects subsequent vicarious pain and/or touch (section B1); six studies instead concern the plasticity of the BOI in vicarious responders (section B2). Finally, seven studies induced an illusion of embodiment over a full-body virtual avatar and investigated subsequent vicarious pain/touch (section C).

**Table 1:** Studies that met the inclusion criteria in the domain of A = vertical desktop-based studies; B1 = body ownership illusion over fake limbs; B2 = body ownership illusion over fake limbs among synaesthetes; C = BOI over full-body virtual avatars. For each study, we report the direction of the effects (increase, ↑, decrease, ↓)

Study	Sample size (N females) Mean age ± SD (or SEM)/age range	Vicarious somatosensation	Behavioral measures of vicarious somatosensation	Visual perspective and/or body ownership induction	Behavioral measures of body ownership	Effects	Effect size	Physiological and/or neural measures/techniques	Effects	Effect size
**A**										
Bach et al., 2014	48 (34), 20.23 ± 3.14)	Pain and Touch	Tactile detection task	1PP vs. 3PP (180°)	None	↓ RTs for 1PP Pain vs. 1PP Touch, 3PP Pain, 3PP Touch	"Viewpoint" x "Action" x "Object": 0.12 (*n*^*2*^*p*)	None	NA	NA
Bowling et al., 2017	Exp 1: 24 (22), 21.7 ± 8.2 ; Exp 2: 24 (16), 23.2 ± 2.6	Touch	Visuotactile Interference Task	Exp 1: 1PP vs. 3PP vs. Dummy vs. Sponge Exp 2: 1PP only (Dummy vs. Sponge)	None	Exp 1= No difference between 1PP and 3PP; Exp 2= ↑ RTs for 1PP Human vs. Dummy during rSI stimulation	"Task" x "Stimulation": 0.43 (*d*)	Exp 1: Offline tRNS at Bilateral SI; Exp 2: Online tDCS at rTPJ and rSI	NA	NA
Bucchioni et al., 2016	20 (12), 24.3 ± 3.34 (range 20-36)	Pain and Touch	NA	1PP vs. 3PP (180°); RHI	Ownership ratings over the desktop hand; Ownership ratings over the RHI	None	NA	TMS	↓ MEP amplitude for 1PP Pain vs. 3PP Pain at late time of stimulation ↓ MEP amplitude for 1PP Pain predicted higher Own over the desktop hand ↓ MEP amplitude for 1PP Pain predicted higher Syn RHI	"Perspective" x "Time" x "Valence": ND "1PP Pain MEPs" - "1PP Hand Self-Attribution": 0.27 (*R*^*2*^) "1PP Pain MEPs" - "Syn RHI Ownership": 0.25 (*R*^*2*^)
Canizales et al., 2013	20 (11), 25 ± 5	Pain	Pain ratings	1PP vs. 3PP (180°)	None	↑ Pain in 1PP Pain vs. 3PP Pain	ND	SSSR	↑ SSSR inhibition for 1PP Pain vs. 3PP Pain	ND
Galang et al., 2019	32 (25), 19.6 ± N.D.	Pain and Touch	Pain categorization and orientation	1PP vs. 3PP (180°)	None	↓ RTs for 1PP vs. 3PP during the Orientation task	"Task" x "Visual Perspective": 0.65 (*d*)	EEG	↑ N2 for Pain in 1PP vs. 3PP ↑ P3 for Pain in 3PP vs. 1PP	N2 "Visual Perspective" x "Valence" during Pain Categorization: 0.59 (*d*) P3 "Visual Perspective" x "Valence" during Pain Categorization: 0:53 (*d*)
Galang et al., 2021	64(55), 18.8 ± 3	Pain and Touch	Pain categorization	1PP vs. 3PP (180°)	None	No effect of Visual Perspective	NA	EEG	↑ P3 for 1PP vs. 3PP in High Social Power	"Visual Perspective" x "Social Power": 0.82 (*d*)
Kuehn et al., 2013	17 (10), 24.9 (20-31)	Touch	Roughness ratings	1PP vs. 3PP (180°)	None	No effect of Visual Perspective	NA	fMRI	↑ Left Posterior S1 in 3PP vs. 1PP	ND
Kuehn et al., 2018	Exp 1: 16 (16), 26.25 ± 3.87 (range 20-36); Exp 2: 13 (13), (range 20-35)	Touch	Roughness ratings	Exp 1: 1PP vs. Real Touch Exp 2: 3PP (180°) vs. Real Touch	None	No difference between real and vicarious touch; No comparison on Visual Perspective	NA	fMRI (Exp.1-2)	Correlation between felt and observed (both 1PP and 3PP) touch in area 3b	ND
Rigato et al., 2019	18 (11), 20.9 ± 1.7	Touch	Visuotactile Interference Task	1PP vs. 3PP (180°)	None	↓ RTs for 1PP vs. 3PP for anatomically congruent touches	"Perspective" x “Anatomical Congruency”: 1.05 (*d*_*z*_)	EEG	↓ P45 for Touch vs. No Touch only in 1PP and Left Hand Stimulation (anatomical spatial match) ↓ P100 for Touch vs. No Touch only in 3PP and Right Hand Stimulation (specular spatial match)	P45 "Condition" x "Perspective" x "Touched Side" : 1.02 (*d*_*z*_) P100 "Condition" x "Perspective" x "Touched Side" : 1.50 (*d*_*z*_)
Schaefer et al., 2009	10 (7), 27 ± 1.75	Touch	None	1PP vs. 3PP (180°)	None	NA	NA	fMRI	↑ Anterior SI (BA 3a,3b) activation for 1PP Touch vs. 3PP Touch ↑ Posterior SI (BA 2) activation for 3PP Touch vs. 1PP Touch	ND
Schaefer et al., 2012	Exp 1: 12 (6), 26 ± N.D. (range 23–39 years) Exp 2: 14 (7), 23 ± N.D. (range 24–30 years).	Touch	None	Exp 1: 1PP vs. 3PP (90°) Exp2: 3PP only	None	NA	NA	fMRI	↑ Posterior SI (BA 2), SII and Insula for 1PP Touch vs. 3PP Touch ↑ leftSI, leftSII and Insula for 1PP Touch vs. Baseline and Movement close to the hand vs. Baseline ↑ bilateral SI, bilateral SII and Insula for 3PP Touch and Movement close to the hand	ND
Vandenbroucke et al., 2015	57 (75%), 23.68 years ± 4.62	Pain and Touch	Visuotactile Interference Task	1PP vs. 3PP (180°)	None	↑ Tactile detection accuracy in 1PP vs. 3PP No effect of Perspective on the number of vicarious somatosensory experiences	"Perspective": 0.20 (*d*)	None	NA	NA
Vistoli et al., 2016	21 (3), 29.2 ± 7.9	Pain	Pain ratings; RTs for pain ratings	1PP vs. 3PP crossed with CPT (Self vs. Other)	None	↓ RTs in 1PP vs. 3PP No effect of Perspective on the Pain ratings	ND	fMRI	↑ Bilateral TPJ for 1PP, Other vs. 1PP, Self PT	ND
**B1**										
De Coster et al., 2013	20 (20), 19.61 ±1.56	Pain	Pain ratings	1PP vs. Visual-only VHI	Ownership ratings	↑ Ownership for VHI vs. 1PP No difference between 1PP and VHI on Pain ratings	ND	Startle blink reflex; SCR; HR	No difference between 1PP and VHI on physiological measures	NA
Fahey et al., 2019	Exp 1: 30 (30); 21.5 ± 0.9 Exp 2: 30 (30); 21.1 ± 1.3 Exp 3: 32 (32); 23 ± 2.7	Exp 1-2 CT Touch Exp 3 CT vs. Non-CT Touch	Pleasantness Ratings	Visuo-tactile RHI	Ownership ratings	↑ Pleasantness for CT Touch after Syn vs. Asyn and vs. NoTouch RHI ↑ Pleasantness on the RHI for CT Touch vs. Non-CT Touch	"Ownership" x "Time point": 0.21 (*n*^*2*^*p*) "Hand Condition" x "Touch": 0.13 (*n*^*2*^*p*)	None	NA	NA
Giummarra et al., 2010	Group 1: 14 UL-Amputees (2), 50 ± 13:3 years (range: 24 - 79) Group 2: 14 LL-Amputees (2), 62 ± 11.24 years Group 3: 14 Non-Amputees (8), 40.25 ± 13.53 years	Pain	Intensity Ratings	Visual-only RHI	Ownership ratings	Similar intensity for vicarious sensations during the Visual-Only RHI for all groups	NA	None	NA	NA
Pamplona et al., 2022	15 (8), 24.7 ± 3.2	Pain and Touch	None	Visuo-tactile VHI	None	NA	NA	fMRI	↑ Left Anterior Insula and Precuneus for Pain and Touch VHI vs. noVHI ↓ Left PMC and ACC for VHI Pain compared to Touch	Left Anterior Insula "Illusion" x "Time": 0.13 (*n*^*2*^) Precuneus "Illusion" x "Time": 0.17 (*n*^*2*^) Left PMC "Illusion" x "TVS" x "Time": 0.23 (*n*^*2*^) ACC "Illusion" x "TVS" x "Time": 0.14 (*n*^*2*^)
Riečanský et al.,^2020^	Exp 1: Group 1: 20 (11), 20.3 ± 2.0 Group 2: 22 (11), 20.6 ± 1.5 Exp 2: 29 (16), 24.5 ± 3.9	Pain	Target-related Painfulness Ratings Observer-related Unpleasantness Ratings	Exp 1 (Behavioral): 1PP vs. VHI Exp 2 (EEG): Ingroup/Outgroup VHI	Ownership ratings	↑ Exp 1: Ownership for VHI vs. 1pp ↑ Exp 2: Ownership for Ingroup vs. Outgroup Exp 2: No relationship between Ownership and Vicarious Pain ratings	Exp 1: "Setup" = 0.19 (*n*^*2*^*p*) Exp 2: "Ethnicity" = 0.48 (*n*^*2*^*p*)	EEG (Exp 2)	↑ mu ERD for Pain during VHI vs. 1PP (EEG data on 1PP setup taken from Riečanský et al.,^2015^) ↑ mu and Beta ERD during stimulus approach associated with Ownership over the virtual hand ↑ Beta ERD during Ingroup vs. Outgroup observation	VHI vs. 1PP: ND "mu ERD Time 1" - "Ownership" correlation: -.34 (*r*) "Beta ERD Time 1" - "Ownership" correlation: -.41 (*r*; negative values indicate a stronger ERD for pain vs. no-pain conditions) "Ethnicity": 0.16 (*n*^*2*^*p*)
**B2**										
Aimola Daivies et al., 2013	MTS: 2 (2), 23-19 Control Group Exp 1: 12 (11), 21.5 ± 2.97 (range 18-28) Control Group Exp 2: 12 (9), 20.7 ± 3,31 (range 18-29)	Touch	MTS experiences; Touch location and intensity	Visuo-tactile RHI	Ownership ratings; Proprioceptive drift	↑ Touch Intensity, Touch Location, Ownership and Proprioceptive drift for MTS vs. Controls during Visual Only RHI	ND	None	NA	NA
Botan et al., 2018	57 NT (29), 21.88 ± 3.45 (range 18–34); 22 SL VPR (17), 21.6 ± 2.15 (range 18–25); 19 AG VPR (14), 21.53 ± 3.1 (range= 19-33)	Pain	Vicarious pain experiences questionnaire	Visuo-tactile RHI	Ownership ratings; Proprioceptive drift	↑ Proprioceptive drift for SL compared to Controls and AG in the Asynchronous condition ↑ Ownership ratings for SL compared to Controls and AG in the Asynchronous condition	Proprioceptive Drift "Group" x "Stimulus Type": 0.04 (*n*^*2*^*p*) Ownership ND	None	NA	NA
Botan et al., 2021	27 NT (19 women); 20 SL VPR (17 women); 12 AG VPR (11 females)	Pain	Vicarious pain experiences questionnaire	Visuo-tactile RHI; Enfacement	RHI: Ownership ratings; Proprioceptive drift Enfacement: Ownership ratings; PSE	↑ Proprioceptive drift for SL compared to Controls and AG in the Asynchronous Predictable but not Random No differences in RHI Ownership ratings between groups across conditions ↑ PSE for SL vs. AG in the Asynchronous Condition ↑ Disownership ratings for SL vs. AG in the Asynchronous Condition	Proprioceptive Drift RHI: "Group" x "Condition": 0.07 (*n*^*2*^*p*) PSE: "Group" x "Condition": 0.07 (*n*^*2*^*p*) other effect sizes: ND	None	NA	NA
Derbyshire et al., 2013	Exp 1: Not related Exp 2: Total sample 52 (52), 20 ± N.D. (range18–22) 19 VPR Group, 33 Control Group	Pain	Vicarious pain experiences questionnaire	Visual-only VHI	Ownership ratings	↑ Ownership ratings for VPR compared to Controls Asynchronous condition	ND	None	NA	NA
Maister et al., 2013	Control 1 Group: 10 (10), 19.9 ± N.D. Control 2 Group: 10 (10), 20.7 ± N.D. MTS Group: 6 (6), 19 ± N.D.	Touch	MTS experiences questionnaire	Enfacement	Self-recognition	↑ Self-recognition into Other Face after Touch observation, only for MTS	ND	None	NA	NA
Vandenbroucke et al., 2013	Exp 1: Total: 30 (23), 21.87 ± 5.99, (range:18–49). 3 excluded from analysis and remaining divided between: VPR Group: 14 (ND), 21.50 ± 4.16 (range:18–34) Control Group: 11 (ND), 23.27 ± 8.76 (range:18–49) Exp 2: Total: 24 (23), 19.17 ± 1.81, (range:17–23). 4 excluded from analysis and remaining divided between: VPR Group: 13 (ND) Control Group: 7 (ND)	Pain	Vicarious pain experiences questionnaire; Vicarious pain errors;	Visuo-tactile RHI	Ownership ratings	Exp 1: ↑ Vicarious pain errors for VPR vs. Controls (not replicated in Exp 2) Exp 2: No differences of RHI susceptibility (Ownership ratings) between VPR and Controls	ND	NA	NA	NA
**C**										
Fusaro et al., 2016	24 (12), 26.1 ± 5.2	Pain, Touch and Social Touch	Intensity, UnPleasantness Ratings	FBOI; 1PP vs. 3PP (180°)	Ownership ratings	↑ Stimulus Intensity for 1PP vs. 3PP ↑ Unpleasantness for Pain vs. Social Touch and Neutral Touch No effect of Perspective on Unpleasantness ratings ↑ Higher Ownership in 1PP vs. 3PP	ND	SCR, HR	↑ SCR for 1PP compared to 3PP ↑ SCR for Pain compared to Neutral and Pleasant Touch ↑ HRD for Pain and Pleasant Touch Neutral Touch	ND
Fusaro et al., 2019	24 (12), 25.4 ± 3.2	Pain, Touch and Social Touch	Intensity, UnPleasantness Ratings	FBOI; 1PP vs. 3PP (180°) crossed with CPT (Self vs. Other)	Ownership ratings	↑ Stimulus Intensity for 1PP vs. 3PP ↑ Pleasantness for Social vs. Neutral Touch and Pain ↑ Higher Ownership in 1PP vs. 3PP	Intensity: "Perspective": 0.32 (*n*^*2*^*p*) Pleasantness: "Type of Stimuli": 0.70 (*n*^2^*p*) Ownership: "Perspective": 0.63 (*n*^*2*^*p*)	SCR, HR	↑ SCR for Pain compared to Pleasant and Neutral touch ↑ SCR for Self CPT compared to Other CPT	"Type of Stimuli": 0.19 (*n*^*2*^*p*) "Group": 0.11 (*n*^*2*^*p*)
Fusaro et al., 2021	Exp 1 Group 1: 22 (22) 21.57 ± 1.54; Group 2 22 (0) 23.48 ± 3.68 Exp 2 Group 1: 21 (21) 24.05 ± 3.23; Group 2 21 (0) 26.52 ± 3.75	Social Touch	Appropriateness, Pleasantness, Arousal, Erogeneity, Vicarious Touch Ratings	FBOI	Ownership ratings	↑ Higher vicarious touch illusion associated with higher ownership illusion	Exp 1 "Ownership" - "Vicarious Touch" Correlation: 0.46 (*r*) Exp 2 "Ownership" - "Vicarious Touch" Correlation: 0.56 (*r*)	SCR, HR	Exp 1: ↑ SCR in Intimate Areas compared to Neutral Areas when touched by a Female Avatar Exp 2: ↑ SCR in Intimate Areas compared to Neutral and Social Areas when touched by a Female Avatar	Exp 1: SCR Model 0.04 (*R*^*2*^*m*), 0.56 (*R*^*2*^*c*) Exp 2: SCR Model 0.02 (*R*^*2*^*m*), 0.54 (*R*^*2*^*c*)
Gonzalez-Franco et al., 2014	19 (10), 25 ± 4.0	Pain	Threat and Harm ratings	FBOI	Ownership ratings	↑ Higher threat and harm illusion associated with higher ownership illusion	"Ownership" - "Harm Hand" correlation: 0.73 (*r*) "Ownership" - "Body Threat" correlation: 0.48(*r*)	EEG, EMG	↑ P450 (C3-CP3), RP (C3-C4) and mu ERD (C3-CP3) for Pain vs. NoPain No EMG differences	ND
Harjunen et al., 2022	58 (35), 26.21 ± 6.44 (range = 18-52)	Pain	Pain, Unpleasantness Ratings	Ingroup/Outgroup FBOI	Ownership ratings	No difference on Pain Ratings during Black and White FBOI	NA	EEG	↑ Beta ERD for Black Pain in Black vs. White FBOI	"Treatment" x "Body Transfer" = 0.085 (*n*^*2*^*p*)
Mello et al., 2022	Exp 1 21 (0), 26.53 ± 3.92 (range = 18–36) Exp 2 21(21), 26.53 ± 3.92 (range = 18–36)	Social Touch	Appropriateness, Pleasantness, Arousal, Erogeneity, Vicarious Touch Ratings	Same/Opposite Sex FBOI	Ownership ratings	↑ Higher Ownership associated with higher erogeneity for touch delivered by a Female avatar in Intimate Areas when men embodied a same and opposite-sex body ↓ Higher Ownership associated with lower pleasantness for touch delivered by a female avatar in Intimate Areas when women embodied a same-sex body	Exp 1 "Same-sex Ownership" - "Erogeneity for Opposite-sex Intimate Touch" Correlation: 0.46 (*r*) Exp 1 "Opposite-sex Ownership" - "Erogeneity for Opposite-sex Intimate Touch" Correlation: 0.5 (*r*) Exp 2 "Same-sex Ownership" - "Erogeneity for Same-sex Intimate Touch" Correlation: -0.38 (*r*)	SCR, HR	↑ SCR in Intimate and Social compared to Neutral areas	Exp 1: SCR Model: 0.31 (*R*^*2*^*c*)
Romano et al., 2016	21 (9), 23 ± 2	Pain	Pain ratings	FBOI; 1PP vs. 3PP (90°)	Ownership ratings	↑ Higher Ownership in 1PP vs. 3PP No difference on pain ratings	"Viewpoint": 0.3 (*n*^*2*^*p*)	SCR	↓ SCR in 1PP vs. 3PP ↓ SCR in 1PP Normal and Big vs. 1PP Thin and 3PP ↑ SCR for Visuotactile Pain vs. Visual-only Pain	"Viewpoint": 0.38 (*n*^*2*^*p*) "Viewpoint" x "Size": 0.15 (*n*^*2*^*p*) "Stimulus Contact": 0.89 (*n*^*2*^*p*)

NA = not applicable; ND = not declared; 1PP = first-person perspective; 3PP = third-person perspective; RHI = rubber hand illusion; VHI = virtual hand illusion; RT = reaction time; CPT = cognitive perspective taking; UL = upper limb, LL = lower limb; EEG = electroencephalogram; fMRI = functional magnetic resonance imaging; TMS = transcranial magnetic stimulation; MEP = motor-evoked potentials; SSSR = somatosensory steady-response; HR = heart rate; SCR = skin conductance response; EMG = electromyography; VPR = vicarious pain responders; PSE = point of subjective equality

## Literature review

### The role of physical perspective

**Fig. 2:**
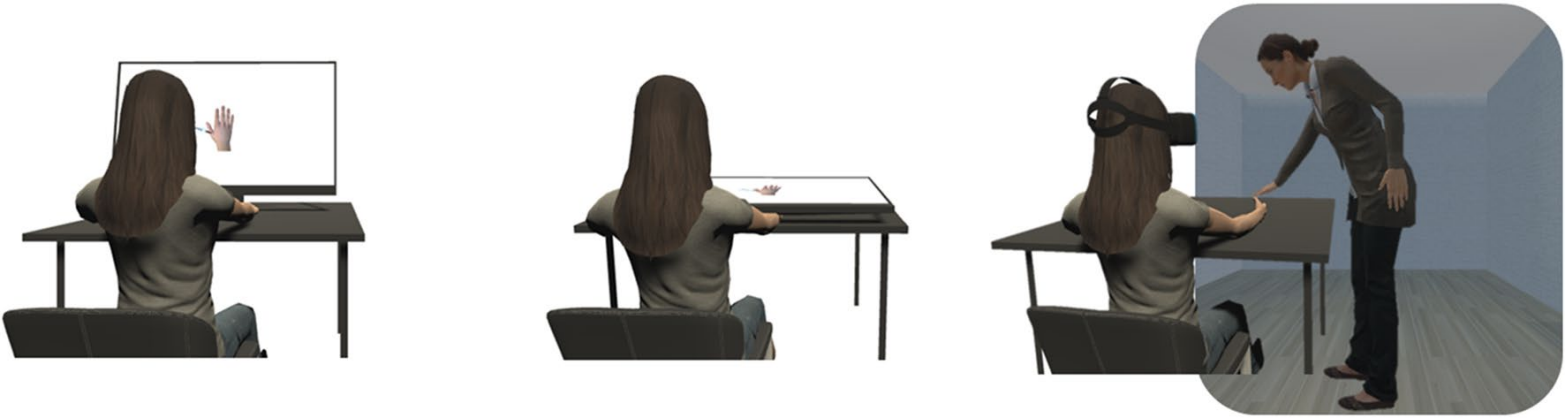
Experimental setups employed to investigate the role of visual perspective and body ownership. First-person perspective (1PP) observation of pain and touch on a desktop, in which the virtual hand is oriented according to an egocentric reference frame (**Panel A**). The illusory sense of ownership over the virtual hand can be increased by placing the screen horizontally, covering the real hand (**Panel B**). An even stronger sense of ownership over a full virtual body can be achieved through Immersive Virtual Reality (**Panel C**). In this case, it is possible to observe from a 1PP somatosensory stimuli delivered on one’s own avatar different body parts by other life-sized virtual avatars

Thirteen studies investigated the role of the orientation of the body parts receiving touch or pain by varying the visual perspective from which the videoclips were presented (Fig. 2A).

Five studies investigated through videoclips the role of physical perspectives on the observation of tactile events. In an fMRI study, Schaefer and colleagues (Schaefer et al., 2009) presented participants with clips depicting hands touched or not by a moving paintbrush either from a first-person (1PP) or third-person (3PP) perspective (rotated by 180°). Both perspectives of viewing touch were associated with somatosensory activation, but the 1PP selectively activated the anterior part of SI (BA 3a, 3b) while the 3PP activated the posterior portion of SI (BA 2). In a subsequent fMRI study, the same authors (Schaefer et al., 2012) compared the vicarious brain activations elicited while observing a touch from a 1PP or 3PP. The 3PP consisted of a hand placed orthogonally to participants’ own hand and facing a second left hand. The authors also considered the possible confounding effects of touch movements occurring in the near-to-hands space. In all videos, the stimulation was delivered on the right hand. Results showed that in 1PP the left SI and SII and the insula were activated, compared to a baseline and a touch received close to the hand. In 3PP, both the touch on the hand or close to it, the bilateral SI, together with SII and insula showed an activation, which in turn was smaller than in 1PP. This pattern of results indicates that the 1PP observation was associated with a stronger and contralateral activation, resembling the activation pattern found for actual touch. However, the observation in 3PP also resulted in a somatosensory activation, pointing towards an important role of SI and SII in processing others’ events. Moreover, the 1PP touch observation was associated with a stronger response in the posterior SI (BA 2) unlike the previous study (Schaefer et al., 2009). The authors interpreted this contrasting finding as a possible effect of the different hand orientation during the 3PP observation, which could have cued different action possibilities and in turn affect vicarious somatosensory response. Kuehn and colleagues (Kuehn et al., 2013) deepened the activation of SI using fine-grained, ultra-high-resolution fMRI at 7 Tesla. They explored both the role of visual perspective (1PP and 3PP) and visual appearance, showing videos of the participant’s or another’s hand being stroked on the index finger by a piece of sandpaper. Similar to Schaefer and colleagues (Schaefer et al., 2009), they found greater activation of posterior SI for the 3PP compared to the 1PP observed touch on another’s hand, suggesting that this portion of SI is selectively involved in the sharing of tactile experiences. However, they did not find a selective activation of anterior SI for 1PP. In a following two-experiments study they selectively investigated whether an anterior part of SI, area 3b, traditionally deemed to be responsible for encoding only felt touch, can be activated also during observed touches (Kuehn et al., 2018) in 1PP (Experiment 1) and 3PP (Experiment 2; 180° hand rotation). Participants observed or felt sandpaper strokes at four locations (ranging from the index to the little finger). They found that 1PP and 3PP touch observation indeed produced a pattern of activation overlapping with the one evoked by felt touches, although weaker in amplitude. Furthermore, they showed that this topographical mapping was selectively active for observed touch and not for observed movements happening close to the hand. It is worth noting, however, that the observed touch from 1PP and 3PP were obtained from two separate samples and there was no within-participants comparison to quantify amplitude differences between the two perspectives. A comparison between the two perspectives was investigated by Rigato and colleagues (Rigato et al., 2019) using EEG. Participants observed videos depicting a left hand touched with a paintbrush, from 1PP or 3PP, and they received a simultaneous tactile stimulation on either the right or the left hand. Therefore, left-hand tactile stimuli matched the video in anatomical coordinates in 1PP and 3PP, while right-hand tactile stimuli matched the video from a specular reference of frame ("mirror-like”) in 3PP. The results showed that an early somatosensory component (P45) was significantly modulated in 1PP when both observed and felt touch occurred on the left hand. A late component, the P100, was instead modulated only in 3PP when participants received tactile stimulation on their right hand and observed touch on a left hand. While the P45 might reflect an early anatomical mapping for touch in 1PP, the P100 might correspond to a later matching of the visual-tactile information in a “mirror-like” specular frame of reference, which is particularly important while observing others.

More evidence on the role of perspectives comes from studies of painful stimuli observation. In a behavioral study, Bach and colleagues (Bach et al., 2014) investigated how the detection of tactile stimuli delivered on the right finger is affected by the observation of painful and non-painful grasping actions from a 1PP or 3PP. The speed of tactile detection is thought to reflect anticipatory activity of somatosensory cortices during action observation, encoding the sensory consequences of the observed actions (Morrison et al., 2013). Participants were faster in detecting tactile targets delivered to their finger when observing painful grasping actions compared to non-painful ones, and this facilitation was selectively induced by the 1PP but not the 3PP observation. By recording motor evoked potentials (MEPs), Bucchioni et al. (2016) investigated the role of visual perspective on sensorimotor inhibition occurring during the observation of painful events on hands from a 1PP and a 3PP (hand model rotated by 180°). Results demonstrated that the inhibition (i.e., freezing effect; Avenanti et al., 2005; Beise et al., 1998) that occurs while observing others’ pain seems to be specific for the observation of the 1PP model, suggesting a major role sense of visual perspective, and as the authors state in their paper, body ownership (i.e., the hand perceived in 1PP) during the observation of painful events. Moreover, the authors conducted an RHI induction in the same participants separated from the MEPs study, in order to obtain a measure of predisposition to experience embodiment: they observed that the stronger the inhibition of MEPs, the greater the embodiment disposition during RHI, supporting their claim about the major role of ownership. However, it is crucial to note that the observed specificity of sensorimotor inhibition to the 1PP model raises concerns about the robustness of the link between sensorimotor empathy and embodiment. Also Vistoli and colleagues (2016) manipulated both the visual (1PP vs 3PP) and the cognitive perspective (i.e., “imagine the hand as your own one vs of another individual”) of the observation of hands receiving painful stimulation. In their research, the authors asked participants to envisage and assess the perceived pain experienced by either themselves or a stranger in a specific scenario. The manipulation of the physical perspective consisted of images of hands viewed either from the participants' own perspective (1PP) or rotated by 180° (3PP). Additionally, prior to observing the stimuli, participants received information about the cognitive perspective: participants were instructed to imagine that the hand displayed on the screen belonged to either themselves or a stranger, irrespective of the physical viewpoint. The results of their study highlighted the interplay between visual and cognitive perspective, which was coupled with an activation of the bilateral temporal parietal junction (TPJ), when hands were presented in 1PP (but not in 3PP), supporting the idea that the visual perspective was an important factor modulating the neural underpinnings related to the cognitive perspective. In 2020, Galang and colleagues (Galang et al., 2020) investigated whether the perception of pictures of painful stimuli in 1PP or 3PP could affect ERP components differently. While observing the images, ERPs were affected at both early and late stages, depending on the perspectives, similar to what has been found by Rigato and colleagues in 2019 (Rigato et al., 2019) during touch observation. The early component N2 was larger during the observation of painful stimuli observed in 1PP probably due to an early processing related to the self. The late component P3 was instead larger for 3PP images, probably reflecting recognition of other’s pain. The authors propose that N2 may signify an initial automatic component signaling the recognition of a similarity between stimuli perceived in others and those perceived in oneself. Meanwhile, the P3 is suggested to denote an awareness of the presence or absence of pain from a third-person perspective. The same paradigm was successively used in a second study by Galang and colleagues (Galang et al., 2021), aiming at investigating the role of social status on the perception of painful stimuli. In conjunction with their first study in 2020, the results suggest that low social status can lead to changes in the cognitive evaluation of other’s pain, as highlighted in the late ERPs. EEG recording was also used by Canizales and colleagues (Canizales et al., 2013) with the aim of measuring cortical responses to non-painful somatosensory stimulation (SSSR) while observing pain. EEG responses were recorded while participants were observing pictures depicting hands in painful or non-painful contexts, from 1PP or 3PP (rotated 180° angle). Behaviorally, images were rated as more painful when observed in 1PP. Moreover, the SSSR was of greater amplitude when images were in 1PP, suggesting that the congruence between the perspective of the observer and the observed image results in an increased somatosensory representation of pain. Prior research has established that witnessing pain or touch on one's own body contributes to improved discrimination (Kennett et al., 2001) and accuracy (Vandenbroucke et al., 2013) in distinguishing actual touch and pain. Building on this foundation, Vandenbroucke et al., (2015) explored whether similar effects could be observed when individuals witnessed touch and pain on others. Participants viewed videos depicting painful, tactile, or no-contact stimuli while concurrently receiving vibrotactile stimulation in either a congruent (matching the location shown in the video) or an incongruent (opposite) position. For instance, in congruent scenarios, if the video displayed a tactile event on the right hand, the participant experienced vibration on their right hand. These videos were presented from either a 1PP or 3PP (video rotated upside-down). Additionally, the authors classified as “vicarious somatosensory experiences” the instances where participants, instead of providing the correct response, reported feeling the vibration in the same location as shown in the video. The findings revealed that witnessing painful events enhanced the detection of vibrotactile stimulation, suggesting that the heightened sensitivity during vicarious experiences of pain is not solely attributable to the observation of a hand being approached or touched. Although the accuracy of detection was greater in the first-person perspective (1PP), the perspective did not influence the frequency of reported vicarious sensations. Lastly, Bowling and colleagues (Bowling & Banissy, 2017) tested whether modulating the cortical excitability of bilateral somatosensory cortex via offline high-frequency transcranial random noise stimulation (tRNS) induces conscious vicarious experiences and alters visuotactile detection among neurotypical participants, and the extent to which the effect of the stimulation can be specific for a touch to a human hand (vs. an inanimate object) and for the visual perspective (1PP vs. 3PP). They used a visuotactile interference task, in which participants had to indicate the location of a tactile stimulus on their own body while simultaneously observing another person (or an inanimate object) being touched in a congruent or incongruent position. The observation of human touch per se, regardless of the perspective, induced greater conscious vicarious feelings compared to inanimate touch. Neither visuotactile detection nor MTS-like experiences were affected by tRNS. In a second experiment, they delivered online transcranial direct current stimulation (tDCS) over the rTPJ and rSI while participants were observing from a 1PP either a human or a dummy hand being touched. They didn’t find an effect of rTPJ stimulation, while rSI stimulation resulted in a modulation of visuotactile detection during the 1PP human touch, replicating previous evidence (Bolognini et al., 2013). It is worth noting that differences in rTPJ stimulation montage (Santiesteban et al., 2012) and a lack of a 3PP condition inducing a strong demand for self-other distinction might account for the null results regarding rTPJ.

### Vicarious pain and touch over embodied fake body parts

#### The influence of multisensory congruency during the body ownership induction on subsequent vicarious pain and touch

A series of studies investigated how BOI over fake limbs can affect vicarious pain and touch and how individual differences in vicarious processing are associated with atypical susceptibility to BOI. In a behavioral study, Fahey and colleagues (Fahey et al., 2019) investigated how different degrees of BOI affect the perceived pleasantness of a subsequent observed touch. After the RHI synchronous or asynchronous blocks, the experimenter performed either CT-optimal strokes (delivered at ~3 cm/s) or neutral touches (tapping gestures) on the fake hand. The results indicated that the reported perceived pleasantness of the observed touch was similar to the perceived pleasantness of the actual touch on the real hand, only after the RHI induction via synchronous stimulation. Crucially, this effect was specific for CT-optimal touch but not neutral touch. Pamplona and colleagues (Pamplona et al., 2022) used fMRI to investigate how different degrees of BOI affect the spatiotemporal brain responses to vicarious pain and touch. The authors used a modified version of the RHI, in which participants observed via MRI-compatible goggles a paintbrush stroking synchronously or asynchronously the right index finger of a virtual hand. After the Virtual Hand Illusion (VHI) induction, participants observed the virtual hand being touched by a syringe plunger (touch stimuli) or a needle (painful stimuli). Firstly, they found greater activation in the premotor and anterior cingulate cortices during late stages of vicarious touch but not pain processing, only when body ownership was boosted by visuo-tactile stimulation. Secondly, after the VHI induction, they found enhanced activation of left TPJ during vicarious pain but not touch. Interestingly, insula and precuneus were activated by both pain and touch, only after the VHI induction. This result implies that these brain regions might play a role in encoding a generalized effect related to the sense of ownership in the experience of vicarious pain and touch. Essentially, the activation in these areas may be associated with the overall perception of ownership and embodiment in the observed virtual hand's experiences, whether it involves touch or painful stimuli.

The modulation of vicarious pain and touch in modified, visual-only RHI paradigms has also been reported. De Coster et al. (2013) placed the computer screen horizontally, covering the participant’s real hand (Fig. 2B), and contrasted this condition with the traditional vertical screen setup. Participants underwent an action imitation phase, in which their movement was imitated by the virtual hand, followed by a pain perception phase, in which the virtual hand was hit by a hammer. Overall, behavioral (self-report measures regarding the experience of pain) and physiological measures (startle blink reflex, skin conductance responses and heart rate) show that being imitated enhances the affective resonance with others. Although BOI was higher in the horizontal screen condition, no differences in pain perception were found between the horizontal and vertical screen setups, suggesting that embodiment did not play a role in the affective responses of observed pain. The comparison between a vertical and horizontal screen setup was also explored in an EEG study by Riečanský et al. (2020). Caucasian participants could observe either ingroup or outgroup hands penetrated by a needle or touched by a cotton swab. Results showed that painful events were characterized by a stronger suppression of the oscillatory activity in mu and beta bands over central electrodes. Interestingly, the activation for both ingroup and outgroup pain was enhanced when the stimuli were presented on the horizontal screen. Moreover, differences in ownership illusion over the virtual hand were correlated with the strength of mu and beta suppression.

#### Body ownership among mirror-touch and mirror-pain synaesthetes

The extent to which plasticity of body representations is involved in the conscious vicarious processing of pain and touch has been investigated by exploring the susceptibility to BOI over body parts in vicarious responders.

Giummarra et al. (2010) investigated the degree of vicarious sensations of pain and touch in a visual-only RHI paradigm in upper-limb amputees, lower-limb amputees, and non-amputees. The rubber hand was passively observed through a mirror in the same perceived body space of the hidden (or phantom) target hand. Although upper-limb amputees were expected to show a more malleable plasticity of body ownership, participants of all groups reported illusory feelings of pain and touch over the fake hand. The results suggest that the mere visuo-proprioceptive congruence between the real and fake hand could be sufficient to elicit vicarious sensations of pain and touch over the fake hand. Aimola Davies et al. (2013) compared the RHI responsiveness of MTS and control participants, across three conditions: synchronous and asynchronous stroking, and a visual-only condition (in which the rubber hand was stroked while there was no stroking on the real hand). Only MTS participants reported the visual capture of touch in the visual-only RHI condition, while control participants did not. The setup of this study was slightly different from that of Giummarra et al. (2010). Here the dummy hand was presented 15 cm far from the position of the hidden hand, suggesting that the perceptual overlap between the viewed location of the fake body part and the perceived location of one’s own body might be the necessary condition to elicit vicarious sensations of touch over the RHI in non-synaesthetic individuals.

Maister et al. (2013) tested whether observed touch among MTS elicits changes in the mental representation of the self, by comparing the susceptibility to the enfacement illusion between neurotypical and MTS participants. The authors used a modified version of the paradigm, in which participants observed another unfamiliar individual’s face being touched, without receiving a touch on their own face. Only MTS participants showed a greater tendency to self-attribute other faces, after observing the other’s face being touched. The results suggest that among MTS people, touch observation is not only associated with conscious tactile perception but also with the inclusion of others in their own face representation, blurring self-other boundaries as described in the experiment.

Four studies focused on body ownership plasticity among vicarious pain responders. Vandenbroucke et al. (2013) assessed vicarious experiences of pain in students, exploring the role of dispositional empathy, hypervigilance to pain, and susceptibility to the RHI. Participants were categorized into vicarious pain responders or control group and observed videos of painful events while receiving occasionally painful sensations in the same or different spatial location (visual/painful stimulus on the left or right hand). The main point of the study was the measure of errors during the observation of other’s pain: it was considered an error when participants reported a pin-pricking sensation in the same location of the visual stimulus during incongruent trials (when the visual stimulus was observed on the opposite hand) or trials with no electrocutaneous stimulation. Vicarious pain responders showed more vicarious errors compared to non-responders in detecting the moment in which they were experiencing the painful sensations. However, this result was not confirmed in their second study, together with no influence of empathy, hypervigilance to pain, and susceptibility to the RHI. Similarly, Derbyshire and colleagues (2013) compared the RHI responsiveness between vicarious pain responders and control participants. Vicarious pain responders reported a higher feeling of ownership over the rubber hand and feeling of touch over it compared to control participants, and these sensations were equally strong both in the synchronous and the asynchronous conditions. Botan and colleagues (2018), investigated whether the pattern of atypical body ownership is specific to subgroups of vicarious pain responders. Using a self-report questionnaire assessing the frequency and the spatial precision of vicarious pain experiences, they identified two subgroups of vicarious pain responders, namely the sensory-localized and the affective-general: the former includes those who feel pain when seeing others in pain in the same body part, while the latter includes those who report a generalized feeling of pain. The authors compared the groups’ susceptibility to the RHI. Only sensory-localized participants reported the illusion of ownership and the proprioceptive drift toward the dummy hand in the asynchronous condition. In a following study, Botan et al. (2021) aimed at clarifying the computational mechanisms beyond the atypical BOI in sensory-localized vicarious pain responders. They manipulated the predictability of the asynchronous stimulation by comparing the strength of the illusion between asynchronous alternating (predictable) and asynchronous random stroking. Results showed that sensory-localized vicarious pain responders feel BOI in the asynchronous condition only when tactile stimulation is predictable. This effect has been proposed as a possible difference in the attribution of causality to the sensory events, according to which vicarious responders are more likely to infer common causes of different sensory signals over larger temporal windows.

### Vicarious pain and touch and ownership of full-body avatars

Research focused also on BOI not only over single body parts but also fake humanoid bodies employing the FBOI in IVR (Fig. 2C). Specifically, seven studies investigated vicarious pain and touch during the FBOI in IVR.

Gonzalez-Franco et al. (2014) combined IVR with EEG to measure the electrocortical signatures of pain perception from a 1PP. They presented participants with a virtual body in 1PP seated on a chair with the virtual right hand placed on a table. In the pain condition, the virtual hand was attacked by a knife, while in the control condition, the knife reached the table. The pain condition led to greater P450 amplitude and a greater event-related desynchronization in the mu frequency compared to the control condition. In line with Riečanský et al. (2020), the threat also led to a greater desynchronization in the mu frequency, an index of motor activation that has been interpreted as motor preparation to avoid the virtual stabbing. It is important to note that electrophysiological signals P450 and mu event-related desynchronization were correlated with the subjective rating of ownership illusion, suggesting that the magnitude of electrophysiological activation following painful observation was associated with the sense of owning the virtual hand.

Using a similar paradigm, Fusaro and colleagues (2016) presented participants with a 1PP virtual body and another one placed in front of the participant and thus seen from a 3PP. Participants saw a syringe, a caress, or a ball reaching their virtual hand or the other avatar’s hand. After each stimulus, they had to report their sensations through three separate visual analogue scales in the virtual scenario: (un)pleasantness was reported on a scale in which numbers from 0 to 49 represented an unpleasant sensation, 50 a neutral one, and 51 to 100 a pleasant one; intensity was reported on a scale in which 0 represented no intensity and 100 maximum intensity; ownership was reported on a scale in which 0 was the absence of embodiment and 100 a maximum sensation. Intensity reports were higher and SCR was larger for the stimuli observed from a 1PP compared to a 3PP. Moreover, unpleasantness reports were higher and SCR was larger for the syringe compared to the caress and the ball stimulus, regardless of the physical perspective. The findings indicate both a qualitative similarity, as evidenced by a consistent pattern across the three conditions, and a quantitative distinction between the two perspectives, with heightened responses observed in the 1PP condition. This pattern of results supports the notion that a shared neural system is engaged in processing stimuli, whether experienced on oneself or observed in others. Romano and colleagues (2016) investigated the role of body width and visual perspective on pain processing by presenting a virtual body from a 1PP or from a 3PP (with a 90° rotation angle). Participants observed painful injections on the avatar’s leg, and in half of the trials, they received a congruent noxious stimulation on their real leg. While visual perspective did not modulate pain ratings, SCR results showed the opposite trend with respect to previous studies (Fusaro et al., 2016). SCR was indeed reduced for stimuli observed from a 1PP compared to a 3PP. The visual perspective also interacted with the body size, so that observing a normal or a wider body from a 1PP led to a reduced SCR compared to the other conditions, including the thinner body. Contrary to the other reviewed studies, here participants were aware that they would receive an actual painful stimulation in some trials. Thus, during actual pain processing, increased illusory ownership may be associated with analgesic effects. To tell apart the role of the physical and cognitive components of perspective-taking, Fusaro and colleagues (2019) asked participants either to observe the virtual body as if it were their own body or someone else’s body. Taking the other-oriented cognitive stance led to experiencing a higher congruency of the stimulus valence (pain felt as more unpleasant and pleasure as more pleasant) and lower physiological reactivity (SCR) compared to a self-oriented stance. As opposed to the previous study (Fusaro et al., 2016), the virtual caress was rated as more pleasant than the ball touching the hand; tellingly, in the 2019 Fusaro et al. study the caress was indeed recorded with a motion capture suit, introducing a more naturalistic movement compared to a computer-generated animation. Fusaro and colleagues (2021) tested the role of the touched body part and the touching avatar’s gender in modulating the vicarious processing of social touch. Heterosexual (Experiment 1) and non-heterosexual (Experiment 2) participants observed a virtual body lying on a beach chair from a 1PP. The virtual body was caressed by a female or a male avatar in different body parts, including taboo ones. The touching avatar’s kinematics were based on the motion capture of a real actor. Touches on areas categorized as social (hand and forehead) were rated as more appropriate and pleasant compared to touches on the neutral (knee and foot) and intimate (genitals and chest) ones. These results were influenced not only by the body part on which touch was delivered but also by the gender of the touching avatar, and the gender and sexual preference of the participants (maximal erogeneity for the avatar that matched the participant’s sexual preference). A positive correlation between the feeling of ownership over the virtual body and the illusion of being touched was also found, suggesting that the higher the BOI, the higher the illusion of being touched. Taken together, the results suggest that the affective processing of observed social touch is dependent on the embodied body part that is stimulated. Mello et al. (2022) investigated how the type of embodied virtual body shapes the feelings of touch pleasantness and erogeneity. In two sessions, participants observed from a 1PP either a gender-congruent or -incongruent virtual body. The gender body swap was associated with a switch in the participants’ perceptions of pleasantness and erogeneity, and this body swap effect was stronger in men than in women*.* Moreover, a higher feeling of ownership predicted a higher feeling of touch erogeneity, although no effect on implicit and explicit sexism measures was found. This study suggests that type of BOI influences the affective evaluation of the vicarious stimuli, since assuming the physical characteristics of the other gender can induce a remapping of the vicarious touch appropriateness and erogeneity sensations onto the other’s gender. In a combined IVR-EEG study, Harjunen and colleagues (2022) investigated whether experiencing ownership over black-skinned hands can enhance Caucasian participants’ vicarious resonance to another outgroup avatar in pain. Participants underwent a BOI induction phase in which they experienced visuomotor synchrony over white or black-skinned hands observed from a 1PP. Subsequently, they observed another (ingroup or outgroup) virtual avatar in front of them receiving either painful injections or cotton swab touches. The authors found an amplified beta event-related desynchronization in response to another black avatar in pain when participants were embodying black hands, thus suggesting that increasing self-other similarity via illusory ownership can boost sensorimotor resonance to another’s pain. It is worth noting that in this study the 1PP hands were not attached to a full-body avatar, a condition which may have diminished the BOI (Tieri et al., 2015).

## Discussion

The recruitment of brain areas involved in first-hand somatosensory perception allows us to understand the somatosensory experience of others, providing a foundational layer of empathy. Here we systematically reviewed the available studies investigating the extent to which vicarious pain and touch can be altered increasing bodily self-other overlap through the manipulation of visual perspective and body ownership.

Concerning vicarious touch, only two studies investigated through EEG the observation of touch in the first-person perspective (1PP), highlighting that it is associated with a modulation of early somatosensory (Rigato et al., 2019) and affective (Galang et al., 2020) components compared to the third-person perspective (3PP). Considering the small number of investigations, further studies are needed to support this evidence. With respect to a functional segregation within the primary sensory cortex (SI) according to the visual perspective, fMRI studies initially suggested that the anterior part of SI might be selectively activated for coding vicarious touch on oneself, while the posterior part seems to be involved in the observation of touch on others (Kuehn et al., 2013; Schaefer et al., 2009). Nevertheless, other studies did not consistently support this hypothesis and rather pointed to the role of other brain areas (such as the insula) in differentiating self from others during vicarious pain and touch. Indeed, besides the modulation of activity within somatosensory cortices, the reviewed studies indicate that the 1PP observation might be associated with stronger activation in areas involved in affective processing. Schaefer and colleagues (2012) found stronger insular activation for touch observation in 1PP compared to 3PP. Similarly, the induction of the Body Ownership Illusion (BOI) over a virtual hand through synchronous visuotactile stimulation was associated with insular and precuneus activation during subsequent vicarious pain and touch (Pamplona et al., 2022). The embodied condition might induce stronger self-referred vicarious feelings, which are reflected in a higher activation of areas involved in interoception (i.e., the sense of the internal states of the body; Monti et al., 2022). A further step could be taken by comparing the neural signatures of observed touch on an embodied avatar with touches on an avatar observed in 3PP in a combined Immersive Virtual Reality (IVR)-fMRI study.

Overall, converging evidence suggests a heightened physiological activation (skin conductance responses, early ERPs components) for vicarious observation in 1PP (Fusaro et al., 2016; Galang et al., 2020), which might indicate a higher salience attributed to the stimuli, supporting the view that “*the body I own is the one I care the most about*” (Bucchioni et al., 2016). It is worth noting that the direction of this effect can be reversed when a concurrent painful sensory stimulation on the real body is expected (Romano et al., 2016). In this case, the 1PP can lead to a reduced physiological response compared to the 3PP, suggesting that owning a virtual body during pain processing may induce analgesia.

Concerning synaesthetes, the reviewed studies showed that both mirror-touch and mirror-pain synaesthetes are more likely to infer BO over fake body parts than neurotypical participants, even in the absence of synchronous visuotactile signals (Aimola Davies & White, 2013; Botan et al., 2018, 2021; Derbyshire et al., 2013; Maister et al., 2013). The atypical BO seems to characterize specifically those who report localized sensory vicarious experiences and not those reporting general affective sharing, supporting the relevance of a shared anatomical mapping in mirror-sensory synaesthesia (Ward & Banissy, 2015). Recent studies suggested that synaesthetes’ malleability in self-other distinction can be also explained at the computational level as an enhanced prior probability to attribute different sensory signals (visual, tactile, proprioceptive) as arising from a common source (Botan et al., 2018, 2021). This view is in accordance with a probabilistic, rather than an all-or-nothing, definition of BO (Romano & Maravita, 2019) that can be affected not only by the multisensory congruency of exteroceptive information but also by the individual’s internal models (Chancel, Ehrsson, et al., 2022). The brain center that might be responsible for computing probabilistic sensory information about BO is the posterior parietal cortex, which is already extensively known to be a fundamental node for integrating information coming from different sensory modalities (Chancel, Iriye, et al., 2022). Further investigations are needed to test whether priors concerning multisensory integration might also account for individual differences in vicarious perception among neurotypical participants. Notably, research has demonstrated that individuals with synesthesia possess heightened skills in mental imagery – the ability to construct a “mental picture” (Marks, 1973) across diverse domains (Barnett & Newell, 2008; Spiller et al., 2015). This enhanced capacity could potentially contribute to their heightened conscious perception of others' pain and touch. However, it's crucial to note that no studies that met the inclusion criteria for this review controlled for the influence of mental imagery. Exploring the role of mental imagery as a potential mediator in the vicarious experience of touch and pain, among both synesthetes and neurotypicals, could yield valuable insights and warrants further investigation. Another potential limitation in the current body of studies lies in the absence of explicit instructions for participants regarding the stance from which they should assess the stimuli. For instance, when participants are asked to evaluate whether a stimulus is perceived as pleasant or unpleasant, researchers typically fail to specify whether they are inquiring about the individual's personal perception or the perceived perception of others – such as "do you find it pleasant?" versus "how pleasant do you think the other person perceives the stimulus?" We reported two studies (Fusaro et al., 2019; Vistoli et al., 2016) that showed how cognitive perspective-taking significantly influences the interaction between visual perspective and the perception of both painful and pleasant stimuli. Further studies should endeavour to state explicitly if participants have to rate the stimuli considering their perspective (observer) of the one of the other (receiver).

Several studies focused on the motor consequences of egocentric processing of vicarious pain. Two opposite trends were found: Firstly, the 1PP perspective has been associated with motor inhibition (Bucchioni et al., 2016), in line with previous research on first-hand pain indexed by TMS motor evoked potentials (Farina et al., 2003). Secondly, increasing the visuospatial overlap with the model by placing the screen horizontally so as to hide the real hand or via a head-mounted display seems to be associated with motor preparation rather than inhibition during pain observation (González-Franco et al., 2014; Riečanský et al., 2020). This latter effect, indexed by mu and beta event-related desynchronization in the sensorimotor regions, has been interpreted as increased readiness for a defensive motor reaction or a tendency to escape. One proposed explanation for this divergent pattern is that corticospinal inhibition reflects the sensation that the pain stimulation cannot be avoided (De Coster et al., 2014). The motor consequences might therefore be task-dependent and affected by the sense of agency over the hand. An open question for future research, therefore, regards the role of the sense of agency over the virtual limb in affecting subsequent vicarious pain (Villa et al., 2022).

Overall, it is important to take into account that methodological differences in the BOI induction are associated with different levels of BOI, which may, in turn, substantially affect subsequent vicarious pain and touch. There is a significant decrease in the BOI when the fake hand is far from the real hand, outside the peripersonal space (Lloyd, 2007). Previous research showed that BOI is disrupted when the fake hand is presented at a distance greater than 30 cm and without synchronous visuotactile stimulation (Costantini & Haggard, 2007), as in the case of desktop-based studies that presented the target model in front of the participant. A stronger sense of BOI can be induced when the participant is immersed in a virtual environment and the whole field of view is covered by a head-mounted display. In this setup, the mere vision of a life-sized virtual body located in the same position of the real body is sufficient to induce the Full Body Ownership Illusion (FBOI; Maselli & Slater, 2013). Considering pain perception, the 1PP allows us not only to understand the mechanisms beyond pain perception without delivering actual sensory pain but also to investigate the potential analgesic role of vicarious touch (Fusaro et al., 2022). Moreover, changing the appearance of the embodied virtual body (i.e., making the participant assume the perspective of another character) might result in modulation of the sensations evoked by the vicarious stimuli. For instance, the body swap paradigm (i.e., observe in 1PP the body of someone else) also opens the possibility of assuming the invulnerable qualities of the virtual body (Frisanco et al., 2022). In the study by Frisanco et al. (2022) they showed that embodying the avatar of God results in changes in the perception of participants’ limits and capabilities. We speculate that embodying the same avatar while observing vicarious stimuli of pain or touch might also alter their perception.

## Implications for empathy research and applications

Our systematic review, aimed at understanding the modulation of vicarious pain and touch through manipulations of visual perspective and body ownership, carries important implications for social cognition beyond the experimental and clinical contexts. In essence, our findings suggest that the capacity for experiencing pain and touch of others exists on a continuum, with conditions such as mirror-touch synesthesia representing one extreme. This continuum implies that individuals, even those without clinical disorders, may exhibit varying degrees of vicarious somatosensory responses in their daily lives. Understanding that individuals may differ in their propensity for vicarious responses enhances our comprehension of how people perceive and empathize with others' physical experiences in various social contexts. Evidence shows that losing a sense of responsibility (Cui et al., 2015; Koban et al., 2013; Lepron et al., 2015) and obeying orders (Caspar et al., 2020) are associated with less vicarious resonance towards others’ pain, suggesting that our ability to resonate with others' sensations may play a major role in shaping social bonds, cooperation, and conflict resolution. This notion is supported by the evidence that altering somatosensory cortex with brain stimulation (TMS and tDCS) affects prosocial decision-making (Gallo et al., 2018), and mirror-pain synesthetes are more likely to donate to reduce other’s pain, and the vicarious resonance correlates with the donation (Ioumpa et al., 2023).

The results of our review can inform the development of strategic regulation of empathy (Weisz & Cikara, 2021) and address some of the limitations of traditional cognitive perspective-taking training (Teding Van Berkhout & Malouff, 2016), in which participants are required to imagine being the target of the event. Cognitive perspective-taking requires reducing the egocentric bias by inhibiting the self-perspective, and it is partially dependent upon the cognitive abilities of the participants (Drayton et al., 2018). A key advantage of the FBOI is the possibility to change one’s virtual body appearance and directly put the participants “in the shoes” of someone else (Peck et al., 2013). Importantly, participants experience ownership over another’s body regardless of their levels of negative implicit attitudes towards the other’s social group (Farmer et al., 2012), probably because multisensory congruent cues can override the negative social attitudes (Maister et al., 2015). Previous research showed that embodying the victim of verbal aggressions can impact emotion recognition in the perpetrators (Seinfeld et al., 2018) through modifications of the default-mode network activity (Seinfeld et al., 2021) and it can also affect subsequent behavioral tendencies in the obedience to the authority (Neyret et al., 2020). Here the reviewed evidence shows that embodying the physical characteristics of the target can override the ingroup bias on vicarious pain (Harjunen et al., 2022) and lead to assuming the other gender perspective during social touch processing (Mello et al., 2022). While the vicarious perception of social touch is expected to fundamentally contribute to the understanding of the other’s attribution of meaning to touch (Peled-Avron et al., 2016), the study of empathy for the social aspects of touch can be considered to still be in its infancy. Considering that unwanted touch is one of the main components of sexually harassing behaviors and that sexual offenders are characterized by lower empathic abilities (Moore & Mennicke, 2020), future research should explore whether experiencing touch experiences on someone else’s body via FBOI can be effective in reducing tendencies to harassing behavior. Finally, the reviewed findings are also relevant in the context of IVR-mediated social encounters. More and more social encounters are taking place in the metaverse, a shared virtual environment in which people interact via embodied virtual avatars (Dwivedi et al., 2022). Future research should investigate how identification with the virtual body may strengthen negative psychological consequences following 1PP unpleasant vicarious experiences, as suggested by the anecdotal reports of sexual harassment and unwanted touch during virtual interactions (Freeman et al., 2022; Wiederhold, 2022).
